# Hydraulic Joint Function and Osteoarthritis

**DOI:** 10.2106/JBJS.RVW.23.00040

**Published:** 2023-12-15

**Authors:** Michael Beverly, David W. Murray

**Affiliations:** 1 Botnar Research Centre, Nuffield Orthopaedic Centre, University of Oxford, Oxford, United Kingdom

## Abstract

» This review of bone perfusion work introduces a new field of subchondral physiology.
» Intraosseous pressure (IOP) measured through an intraosseous needle reflects conditions only at the needle tip rather than being a constant for the whole bone.
» Measurements of IOP in vitro and in vivo, with and without proximal vascular occlusion, show that at rest, bone is perfused at normal physiological pressures.
» A subtraction perfusion range or bandwidth at the needle tip offers a better measure of bone health than a single IOP.
» With ordinary loads, very great subchondral pressures are generated.
» Subchondral tissues are relatively delicate but are microflexible with bone fat being essentially liquid at body temperature.
» Collectively, the subchondral tissues transmit load mainly through hydraulic pressure to the trabeculae and cortical shaft.
» White vascular marks on normal magnetic resonance imaging scans are present but are lost in early osteoarthritis.
» Histological studies confirm the presence of those vascular marks and also choke valves capable of supporting hydraulic pressure load transmission.
» Osteoarthritis seems to be at least partly a vasculomechanical disease.
» Understanding subchondral physiology will be key to better classification, control, prognosis, and treatment of osteoarthritis.

Bone blood supply was originally explored by dissection, injection, and clearance studies, but all that work has of necessity been under static or morbid conditions^1^. The measurement of bone blood flow in vivo has attracted little attention^2^. If activity itself alters bone or subchondral blood flow, it would not have been visible to previous static or in vitro investigation^3^.

For nearly a century, intraosseous pressure (IOP) has been studied by using intraosseous needles in vivo but surprisingly, never during activity^4-9^. IOP varies but is generally understood to be elevated in osteoarthritis and osteonecrosis and with bone pain^10^. In practice, IOP is of little use clinically, and a “normal” IOP is difficult to define^11^. In patients undergoing forage or decompression for osteonecrosis, pain relief is often dramatic, but IOP measurement bears no relationship to that benefit.

A standardized model to explore IOP pressure confirms great variability in basal IOP measurements. However, for each IOP measurement, there is a proportional pulse pressure (PP)^12^. Because of the variable types of blood vessels struck by the needle tip, each IOP record will be different. That explains why IOP does not have a single fixed value, present throughout the bone and uniform in all subjects.

Denham showed that many times body weight is transferred across joints during ordinary walking^13^. Huge surface pressures, estimated to be up to 18 MPa or 180 atmospheres equivalent to 135,000 mm Hg or 2,600 psi, well over 50 times car tyre pressure have been demonstrated^14^. Those massive forces act on soft cartilage which rests in turn on a delicate subchondral plate, supported by fine bony trabeculae. The trabeculae are themselves surrounded by large thin-walled fat cells and capillaries. Quite how those delicate tissues tolerate enormous loads has not previously been considered^15^.

As orthopaedic surgeons are aware, at body temperature, bone fat is virtually fluid. Bone fat oozes out of fractures and is seen as a horizontal blood-fat fluid level on lateral fracture x-rays. Fat sprays from saws and drills during surgery. While other fatty tissues are soft and greasy but solid, uniquely bone fat is fluid, embolizes and furthermore, is preserved during starvation^16,17^.

Previous experiments considered that bone might be hydraulically strengthened but that work was unphysiological using grease-saturated dried bone or single cubes of cancellous bone^18^.

It has been found that normal water bright T2 or PD_SPAIR magnetic resonance imaging (MRI) images demonstrate axial plane subchondral radiating vascular marks which are quite different to the classically accepted pattern of bone circulation^19^. Those vascular marks are reduced or almost absent in early osteoarthritis^20^. Histologically, the same marks are easily demonstrated and are clearly vascular. Subcortical choke valve–like structures were also identified.

This suggests that load across the joint may be transmitted through the subchondral cancellous bone by a hydraulic mechanism involving liquid fat and blood constrained within the bone by valves^16^. Furthermore, failure of this mechanism is likely to be a key feature of arthritis. This review aims to assemble and enlarge on previous work, exploring these linked observations, to support a better understanding of joint function and osteoarthritis^21^.

## Resting IOP

**Fig. 1:**
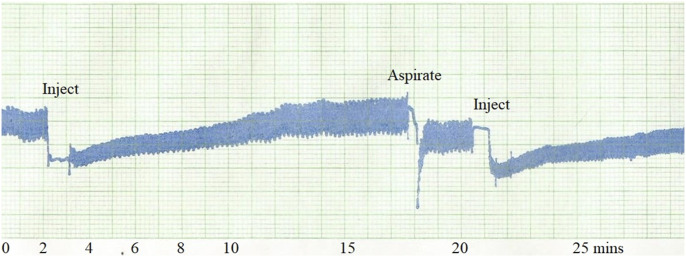
Needle clearance by saline injection reduces IOP with a slow recovery over about 10 minutes. Following clearance by aspiration IOP recovery takes less than half a minute.

When using an intraosseous needle connected to a pressure gauge in vivo in normal subchondral cancellous bone, the initial saline flushing or clearance, “the Ficat technique,” should be avoided. Even a small injection forces saline, heparin, fat, blood, and bone fragments back into the vascular tree, damaging the delicate microcirculation around the needle tip. This causes a persistent fall in IOP for up to 10 minutes as in Figure 1, while after aspiration, recovery in IOP is much faster within about a minute. It is also possible that previous workers may have been measuring a raised IOP because of the saline injection itself into poorly drained or avascular bone^10^.

**Fig. 2:**
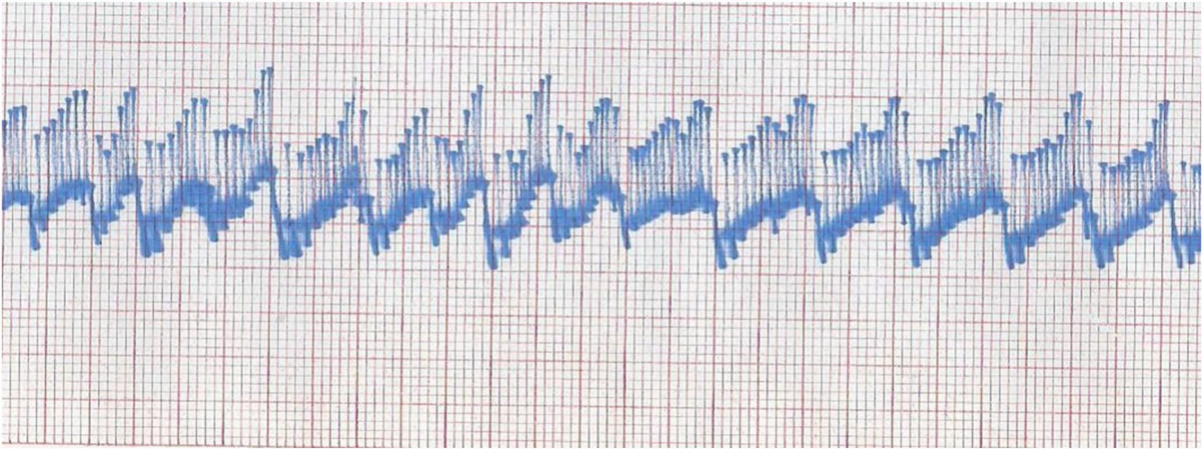
Cardiac and respiratory waves seen in an IOP recording lasting about two minutes.

IOP recordings at rest exhibit fluctuation with the arterial pulse. When the trace is slowed down, there is an underlying wave synchronous with respiration as in Figure 2. Drugs which cause systemic pressure changes can also be followed through an intraosseous needle. The IOP trace shows wave forms corresponding to a circulation time of around 90 seconds^22^.

IOP varies considerably within and between subjects. While IOP is not greatly affected by site or size of the needle, by sex or weight, there is a correlation with systemic blood pressure (p < 0.0005). Furthermore, a correlation exists between any IOP measurement and its associated PP (p < 0.001). This implies that IOP reflects the perfusion conditions only at the needle tip rather than there being a constant pressure throughout the bone^12^.

Arterial occlusion with a proximal clamp or tourniquet causes a reduction in pressure (IOPa) (p < 0.0001) and complete loss of PP while proximal venous obstruction always elevates pressure (IOPv) (p < 0.012) with retention of the PP. Subchondral cancellous bone at rest therefore performs just like any other perfused tissue with IOP being mainly due to arterial supply pressure, rather than being a back pressure or some form of tissue turgor.

It follows that with proximal arterial obstruction, the residual IOPa is in fact the real back or venous drainage pressure. Similarly, IOPv measured during proximal venous occlusion demonstrates the best supply pressure achievable at that needle tip. So while an individual measure of IOP is effectively of no value, being a random measure from vessels encountered at that particular needle tip, simply clamping the proximal vessels alternately, perhaps with a tourniquet, offers a new subtraction method (IOPv − IOPa) for exploring the physiological health or “perfusion bandwidth” of that small area of bone at the end of the needle^23^.

In avascular or poorly perfused bone, irrespective of the starting point or basal IOP, the subtraction difference is small, while in healthy bone, the perfusion range is greater. IOP and PP also depend to a degree on the “clearance” volume created at the needle tip. Once the initial “shock” from a clearance injection has settled, a larger clearance has a greater chance of exposing the needle tip to a larger and therefore higher-pressure vessel. The subtraction technique has not been previously described but may be more widely applicable.

For example, a more logical means of testing for compartment syndromes might be to measure compartment pressure through a needle as usual but then with a proximal arterial pressure cuff in position (Pa). After emptying the limb by elevation, the compartment pressure is measured again but with a venous tourniquet in place (Pv). By subtraction (Pv − Pa), the perfusion range deep in the compartment at the needle tip is obtained. Where the difference is large, there is good perfusion and surgery might be delayed. If there is a narrow range, perfusion is poor and surgical decompression more urgent.

## IOP and Loading

**Fig. 3:**
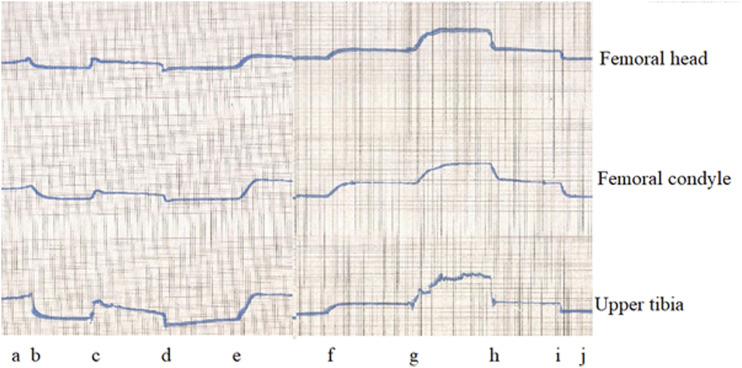
Simultaneous IOP recording from the femoral head (upper), femoral condyle (middle) and upper tibia (lower) traces with (a) basal state, (b) proximal arterial clamp applied, (c) load one body weight applied, (d) load removed, (e) arterial clamp removed, (f) proximal venous clamp applied, (g) load one body weight applied, (h) load removed, (i) venous clamp removed, (j) return to basal state. Pressure range 0–100 mmHg for each trace, total 10 minutes duration.

During loading with and without proximal arterial and venous tourniquets, subchondral IOP changes are similar throughout the limb. A simultaneous recording from the femoral head, femoral condyle, and proximal tibia in the rabbit model shows the changes to be widespread through both bones as in Figure 3. With a 1 body weight load, there is a rise in IOP. The same load during arterial obstruction gives a smaller gain in IOP, but with the same load during venous occlusion, a much greater rise in IOP is found, akin to squeezing a full sponge.

In addition, alternating saline injections at all 3 sites show that while pressure is elevated throughout the length of a bone, it does not cross the knee joint. Long bones therefore act as separate semiclosed compartments, but joints are effectively barriers to pressure transmission. In conclusion, IOP does not have a fixed value but depends on the needle tip vessels encountered. Variations are due to background systemic pressures and hydraulic pressure from loading. Whether perfused or not, load increases subchondral IOP proportionally and instantly.

## Osteonecrosis and Steroids

**Fig. 4:**
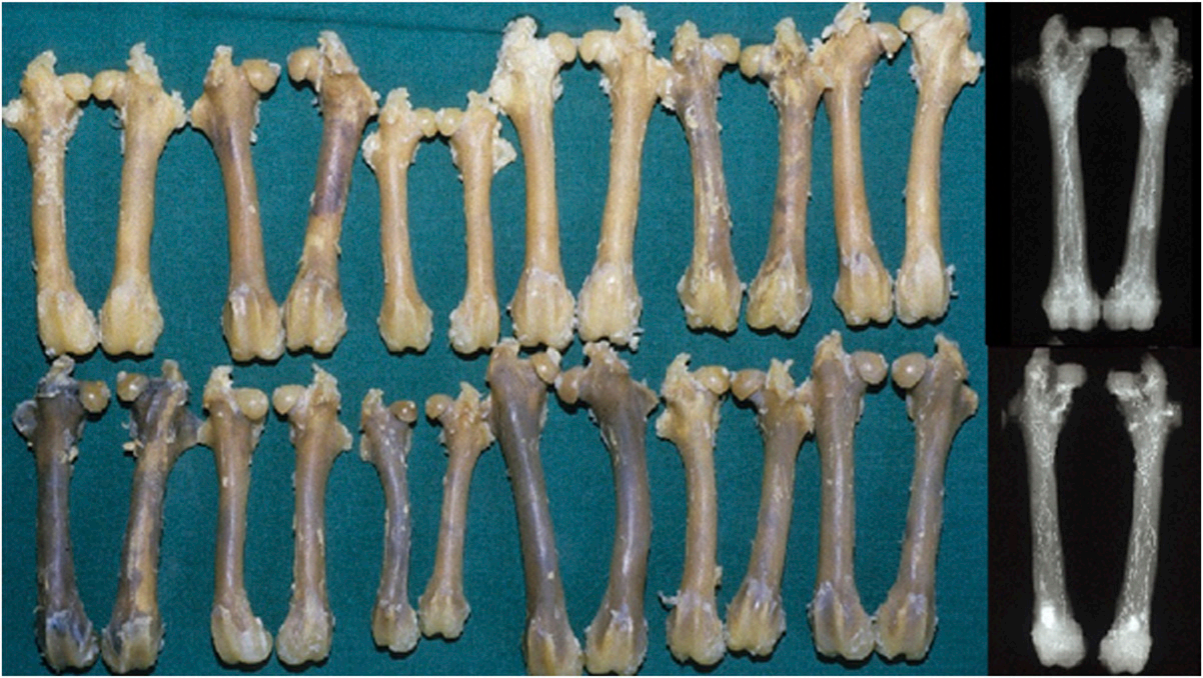
Top row steroid treated, lower row controls. Barium angiography followed by excision and decalcification. Steroid treated bones whiter due to retaining more barium than controls. Example angiograms from each group on right side.

For 50 years or more corticosteroids have been believed to cause fat cell swelling, leading to venous obstruction and osteonecrosis^24-28^. However, recent work shows that in a particular high dose steroid treated rabbit model, there is cachectic weight loss and shrinkage of the osseous fat cells. This is confirmed by angiograms and photographs of the decalcified bones (Fig. 4). The steroid treated specimens have a higher IOP, not because of a raised venous back pressure but because there was a better arterial supply and wider “bandwidth” at the needle tip. Perhaps more excitingly, the work shows that by altering intraosseous volume, it is possible to change intraosseous perfusion^29^. Of course, this does not explain why osteonecrosis is associated with corticosteroids in man, but it does show that IOP and subchondral vascular physiology may be important in understanding bone disease.

## Experimental Bone Loading

Calf feet were collected from male calves culled at about 1 week. The radial artery to the foot was catheterized and perfused with serum allowing for a few hours in vitro IOP measurements with different patterns of loading and vascular occlusion. Loading was applied either with an external fixator or more physiological direct hoof pressure mimicked standing and walking activity patterns^30^.

Initially, because serum is perfused into the foot, there is a slow rise in IOP over up to a minute. This reverses if the perfusion is stopped. Static fixator loading gives an instant and proportional rise in subchondral IOP, whether or not the bone is being perfused. Whatever the perfusion conditions, IOP is proportionate to loading. Furthermore, removal of the load is followed by an instantaneous drop in IOP, surprisingly lower than the preload value. The system is therefore not fully elastic, or the pressure would drop to about the same value. And where “walking” is simulated by repeated hoof pressure, there is a progressive fall in subchondral IOP, with or without perfusion. This indicates that there must be a valve or some other device to assist subchondral circulation during activity^30^.

**Fig. 5:**
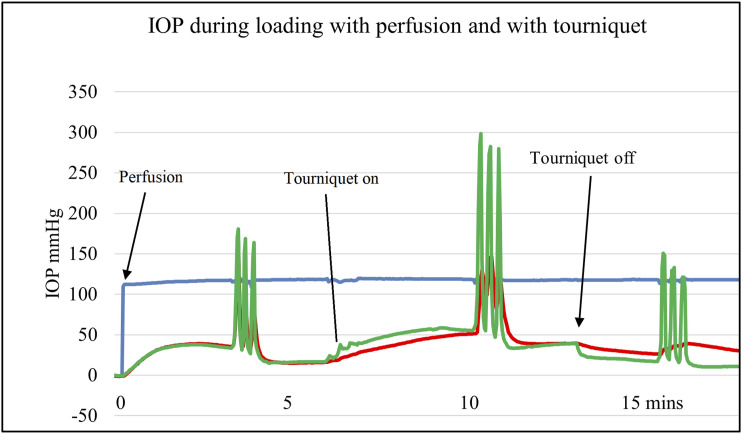
IOP loading and perfusion. Example of IOP with loading during perfusion (left group), with proximal venous pressure tourniquet (central group) and perfusion alone (right group). Blue – serum perfusion pressure, Red – metacarpal epiphysis IOP, Green – metacarpal subchondral IOP. Each set shows the effect of a static load of 10 kg applied for 10 seconds with 10 second rest intervals, x 3. The tourniquet was applied at 6 minutes and removed at 12 minutes (arrows).

Ordinary physiological loads give subchondral IOPs well above systemic circulation or capillary pressure. This indicates that with greatly raised IOP, much of the force is transferred hydraulically through semiliquid fat and other tissue to the trabeculae. Hydraulic pressure therefore plays a part in force transfer to the trabeculae which in turn coalesce toward the cortical shaft^12^ (Fig. 5). Load is carried down along the cortical shaft, and the process operates in reverse at the next joint. There the trabeculae compress fat which in turn supports the microflexible endplate and cartilage.

## Radiology

**Fig. 6:**
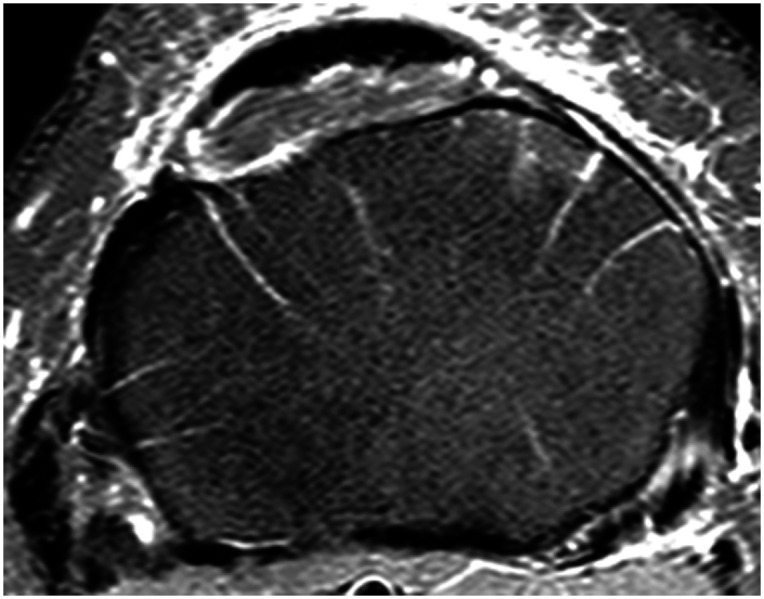
Normal proximal tibial slice from a PD_SPAIR MRI scan showing the radiating water bright vascular marks.

Normal water bright T2 or PD_SPAIR MRI scans exhibit white marks in subchondral axial slices. Despite their frequency, they have not previously been described. Radiological opinion is that they are vascular. In the subchondral axial plane of the femur and tibia, they radiate from the center to the cortex or periphery as in Figure 6. While present in all large synovial joints, they are seen best in the relatively flat upper tibial articulating surface where they lie about 1 cm below the surface. In curved or smaller joints, they are less easily seen. Typically, the white marks begin to appear about 5 mm below the tibial articular surface, increase in number to the 1 cm level, and are lost by 2 cm below the joint surface.

**Fig. 7:**
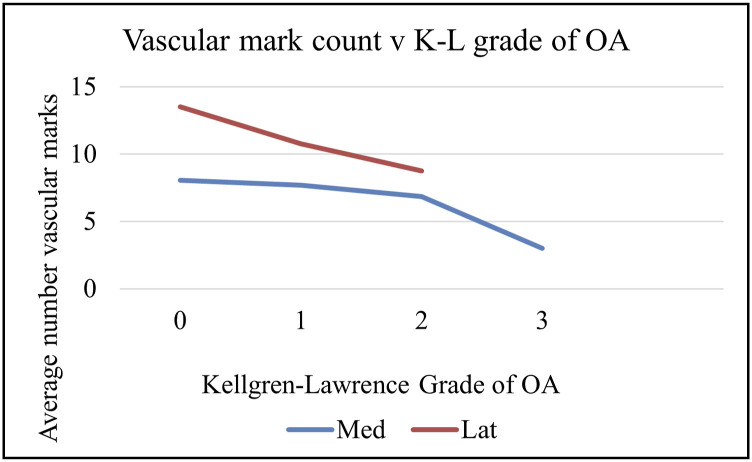
Number of vascular marks in the upper tibial slices with corresponding plain x-ray grade of osteoarthritis on Kellgren-Lawrence scale for lateral (red) and medial (blue) compartments.

Most arthritis in knees begins medially. Osteoarthritis can be crudely scored on the Kellgren-Lawrence (K-L) 1 to 4 scale. When MRI scans from the same patients have the vascular marks counted and compared with the K-L score, there was no correlation with the presence of MRI marks and age, side, sex, or weight to be found, but there is an inverse correlation between the frequency of the marks and the corresponding K-L grade of osteoarthritis both medially and laterally as in Figure 7. It is not clear which comes first or whether one is a consequence of the other. It seems more likely that vasculomechanical failure and osteoarthritis go hand in hand and represent a final common pathway whatever the primary pathology^20,31-33^.

## Anatomy and Histology

**Fig. 8:**
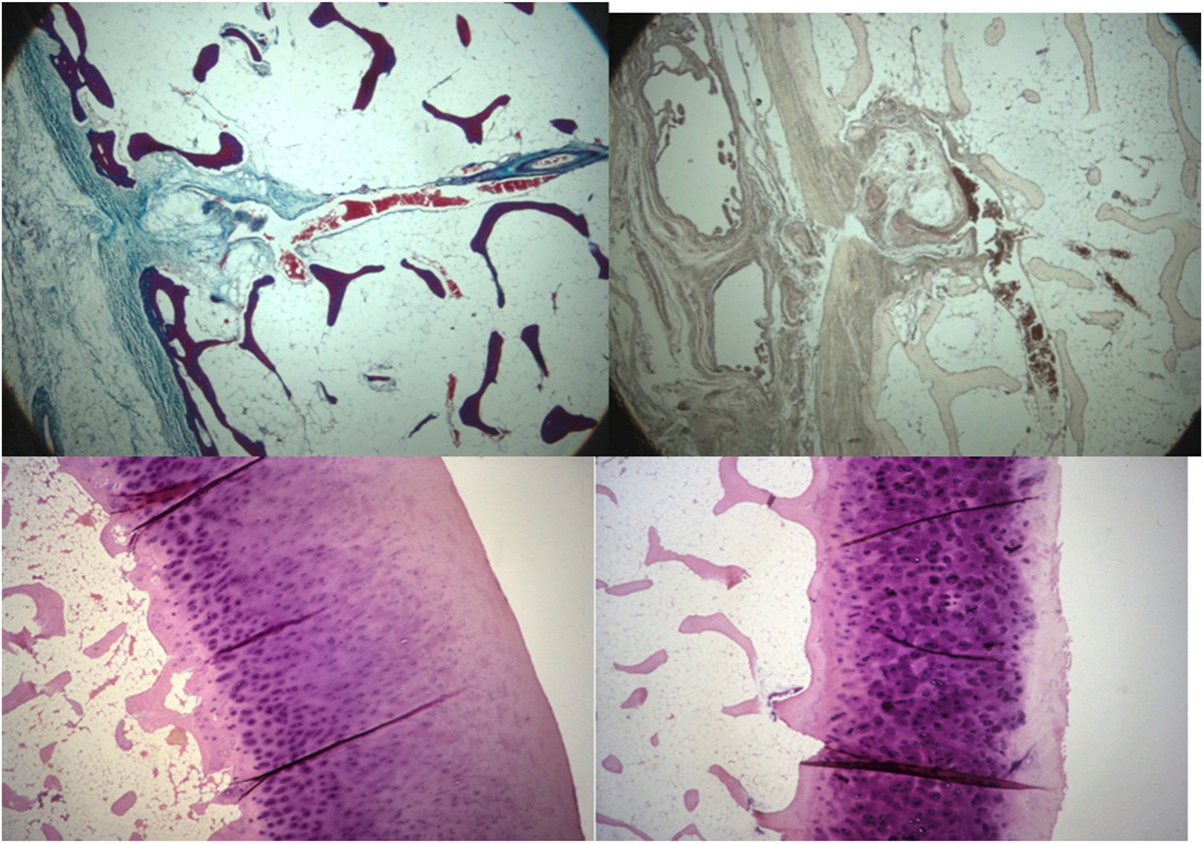
Upper pair: Cortex on the left, example of vessels seen on MRI in the subchondral plane about 1 cm below the tibial joint surface. Vascular distortion or possible choke valve as they reach the cortex. Goldner's trichrome x 20 and Masson's trichrome x 20. Lower pair: Left normal femoral head with plentiful subchondral vessels, right early osteoarthritic femoral head with none. H+E x 20.

If bone is slightly flexible and subchondral adipocyte fat is effectively fluid, for load to be transmitted by hydraulic pressure, one might expect there to be histological or anatomical features to support that role. Nearly 300 years ago, William Hunter found vessels beneath the cartilage (Hunter's mesentery), but since then, they have mainly been ignored^34^. Yet examination of nearly any section through a healthy joint in the sagittal plane reveals a network of many subchondral capillaries just underneath the cartilage endplate. The number of vessels appears to be related to the thickness of the overlying cartilage^16^. Fifty years ago, Burkhardt described choke valve–like cells in cancellous bone capillaries. He also noted muscle cuffs around some periosteal veins^35^. He did not offer a reason for these observations, but they may have a vasculomechanical purpose. The marks on T2 MRI scans are described above and are confirmed histologically to be vascular in normal bone. The same vessels penetrate the cortex close to the articular margin, as seen on any dry bone. Just below the juxta-articular cortex, there are complex vascular convolutions or distortions. Those would act as choke valves closing instantly when the surrounding fatty tissue pressure rises, as with load bearing (Fig. 8, upper).

While lipid does not move or flow under load, the fat is contained in large thin-walled adipocytes and the lipid is oily or liquid at body temperature. Such tissue is able to transfer high pressure, provided that it is in a closed environment. Other fine structures such as the red cells and capillaries equally experience the same enormous pressures, but in a contained space with no capillary flow, there is no turbulence to cause damage. In this beautifully designed system, it can be seen that the subchondral tissues together allow transfer of load, at least partly, but probably mainly, by hydraulic pressure on to the trabeculae. The fine trabecular fan unites onto the cortical shaft, passing the load down the shaft to the next joint. There the opposite process takes place with the load moving onto the trabeculae and into the microflexible fatty subchondral tissues. There the same great pressure is generated, in turn supporting the cartilage endplate and the joint surface. The falling IOP with repeat activity, irrespective of the perfusion state, indicates that there must also exist some form of valve, such that activity promotes circulation. The whole design allows circulation to continue at normal physiological pressure once the load is removed. Furthermore, it seems that in early osteoarthritis, the number of subchondral vessels is reduced, suggesting vasculomechanical failure and subchondral sclerosis as the cause or final common pathway in the generation of osteoarthritis^16^ (Fig. 8, lower).

This potentially offers a vasculomechanical explanation for the nearly mutually exclusive nature of osteoporosis and osteoarthritis. There is usually healthy cartilage in a fragility fracture of the femoral neck whereas the arthritic hip seldom fractures. The thinner more flexible bone of the porotic patient flexes more and is therefore better perfused than the stiffer more avascular bone of the arthritic. Poor subchondral perfusion leads to loss of cartilage nutrition and secondary cartilage failure. Although surface fibrillation is almost the first visible sign of early osteoarthritis, it is probably subchondral ischemic sclerosis with a loss of flexibility and reduced vascular perfusion that is the primary event. Weather-related bone pain or aching may be explained by the falling atmospheric barometric pressure causing relative congestion in precariously perfused subchondral tissue^36-38^. Such aching pain is usually relieved by activity.

## Conclusion

Our review of the physiology of the subchondral region presents a fresh understanding of joint function. While a single IOP measure is of little value, assessing perfusion at the needle tip by a simple subtraction technique is likely to be valuable. Other compartments may be similarly explored. MRI vessel counts offer a means of classification of osteoarthritis. Subchondral tissues are beautifully designed to support the enormous forces of load transmission by hydraulic pressure through semiliquid fat and blood that is held within the bone by valves. A “vasculomechanical” understanding of joint function helps to explain osteoarthritis and some other joint pathology. This fresh approach should lead to further research and eventually better management of osteoarthritis.

### Source of Funding

The work was partly supported by a grant from the Wellcome Trust (12425/1.5/SC) but was mainly self-funded.
